# Editorial: Spotlight on nitric oxide: integrative approaches to study NO and RNS in physiology and disease

**DOI:** 10.3389/fphys.2026.1779024

**Published:** 2026-01-29

**Authors:** Erika M. Palmieri, Andrew Gow

**Affiliations:** 1 Cancer Innovation Laboratory, National Cancer Institute (NCI)-Frederick, National Institutes of Health, Frederick, MD, United States; 2 Department of Pharmacology & Toxicology, Ernest Mario School of Pharmacy, Rutgers University, Piscataway, NJ, United States

**Keywords:** free radical signaling, immunomodulation, nitric oxide, NO, reactive nitrogen species, RNS, redox biology, translational medicine

The simple diatomic molecule nitric oxide (NO), alongside its reactive nitrogen species (RNS) derivatives, remain giants in contemporary physiology and pathology. NO plays many roles, spanning the control of vascular tone, the regulation of muscle function, and the modulation of immune responses and cell growth. A pervasive question in this field is how such a seemingly simple molecule exerts control over such a wide range of biological processes. Critically, what sets NO apart from other signaling molecules is its nature as a free radical, a characteristic that underlies its peculiar reactivity and duality of action, enabling both beneficial signaling and potential oxidative damage depending on the biological context (Win et al., 1998). This Research Topic was conceived to capture cutting-edge research that moves beyond siloed organ studies to embrace the integrative complexity of the NO/RNS network. NO research is at a pivotal moment, reflecting a molecule of elegant biochemical simplicity that nonetheless commands complex and far-reaching regulatory roles in physiology and pathophysiology. This Frontiers collection synthesizes research that not only details the molecular mechanisms by which NO and reactive nitrogen species (RNS) navigate biological signaling but also integrates translational dimensions that will shape the future of therapeutic, diagnostic, and physiological interventions.

At its core, NO serves as a master regulator of cellular communication, with its production tightly controlled by the family of NO synthases (eNOS, nNOS, and iNOS) and alternative pathways including the nitrate–nitrite–NO reductive cycle. These regulatory processes serve to allow for NO generation at the right time for the signaling pathway involved (Thomas et al., 2008; Gow et al., 2004). The regulation of endothelial NO synthase (eNOS) alone exemplifies this complexity. The enzyme’s dimerization, intricate cofactor requirements (e.g., tetrahydrobiopterin BH4, FAD, FMN), and modulation by cellular redox states and protein-protein interactions finely tune NO bioavailability to meet varying physiological demands, such as dynamic vascular tone adjustments during exercise or hypoxia (Thomas et al., 2001). Disruptions in cofactor availability or excessive scavenging by endothelial hemoglobin α-subunits exemplify avenues leading to endothelial dysfunction and vascular diseases (Ignarro et al., 1987; Wink et al., 2001; Kuhn et al., 2017). Additionally, eNOS critically depends on Ca^2+^ for activation (Tejero et al., 2010). Inducible NOS (iNOS) is a Ca^2+^-insensitive form of the enzyme that is typically absent at baseline but is robustly induced in various cells, particularly macrophages, in response to inflammatory stimuli (Espey et al., 2000; Somasundaram et al., 2019). Transcriptionally regulated by inflammatory mediators such as NF-κB and IRF1, iNOS generates high-output NO over sustained periods, underpinning cytotoxic antimicrobial defenses and contributing to tumoricidal activity (Thomas et al., 2015). Regulated at the post-translational level, including by proteasomal degradation and aggresome-mediated sequestration (Pandit et al., 2009), iNOS activity dynamically balances host defense with limiting collateral damage. Its pivotal roles in systemic inflammation and sepsis have inspired pharmacological investigations targeting iNOS for therapeutic modulation across infectious and chronic inflammatory diseases (Wink et al., 2011).​

In contrast, neuronal NOS (nNOS) is constitutively expressed and tightly regulated by intracellular calcium fluctuations, binding calmodulin, reflecting its key roles in neural signaling, synaptic plasticity, and neuromodulation. Beyond the central nervous system, nNOS participates in skeletal muscle contractility and vascular tone modulation, bridging neural and cardiovascular physiology (Stuehr, 1999; Ford and Lorkovic, 2002; Abu-Soud et al., 1995). The divergent calcium dependence and temporal NO release profiles embody the functional spectrum of NOS enzymes, which are tailored to diverse biological imperatives. A key aspect of this spectrum is the flux rate of NO production, as it appears for physiological NO signaling, it needs to be made in the right place at the right time and at the right rate. This foundational knowledge anchors the broader implications addressed by the Research Topic and contextualizes the diverse roles NO plays across tissues.​

One compelling theme is the intersection of NO with cellular metabolism and immunity, as evidenced by contemporary studies of macrophage immunometabolism (Palmieri et al., 2020a; Palmieri et al., 2020b; Palmieri et al., 2023). NO’s concentration-dependent effects orchestrate various cellular outcomes, ranging from low-level signaling to pathological states. This necessitates precise modulation and context-aware investigation. For example, the metabolic reprogramming of inflammatory macrophages illustrates NO’s dual regulatory roles. NO-derived electrophilic nitroalkenes, such as nitro-oleic acid (NO2-OA), sculpt macrophage polarization by repressing inflammatory cytokine production and reshaping metabolic fluxes. By reducing the accumulation of TCA cycle intermediates, such as succinate and itaconate, metabolites known to stabilize pro-inflammatory Hypoxia-Inducible factor (HIF-1α) and drive cytokine secretion, these nitroalkenes unveil non-canonical pathways through which NO derivatives resolve inflammation. This metabolic rewiring extends to altering cellular reliance on glutamine and redirecting it toward glutathione synthesis, thereby enhancing antioxidant defenses. The study by Stevenson et al. applies advanced liquid chromatography-high-resolution mass spectrometry (LC-HRMS) alongside stable isotope (^13^C) tracing of both glucose and glutamine to dissect central carbon fluxes in macrophages upon NO2-OA treatment, directly comparing them with 1400W (a standard NOS2 inhibitor) and oleic acid controls.​ The study also quantifies the formation of NO2-OA–glutathione (GS-OA-NO2) adducts via targeted mass spectrometry, providing insight into cellular thiol alkylation as a direct metabolic and signaling event.​ Importantly, formation and quantitation of GS-OA-NO2 adducts reveal a direct covalent interaction of NO2-OA with cellular GSH pools, connecting lipid-derived electrophiles to antioxidant defense in inflammatory settings.​ Beyond mechanistic insights, the authors’ findings show promise for clinical translation, with nitroalkene-based compounds under phase 2 investigation for chronic pulmonary inflammation and broader inflammatory diseases, suggesting a new class of endogenous-inspired therapeutics that target immune-metabolic communication.

Further linking NO to disease biology, NO’s modulation of the human epidermal growth factor receptor (HER/ErbB) family underscores the complexity of oncogenic signaling nuances modulated by nitrosative signals. Either by direct S-nitrosation or by increasing phosphorylation, NO can influence receptor dimerization and downstream MAPK, PI3K-Akt, and JAK-STAT signaling cascades central to cellular proliferation, differentiation, and survival pathways. These molecular modifications not only contribute to tumor progression but also potentially alter responses to targeted therapies. For instance, the ability of NO to modulate the phosphorylation states of EGFR and HER2 correlates with both enhanced tumor aggressiveness and therapeutic resistance, emphasizing the translational need for combinatorial approaches that integrate NO pathway modulation alongside HER-targeted regimens. The work by O'Neill et al. demonstrates that S-nitrosation of EGFR at C498 directly activates the receptor and downstream oncogenic signaling. This process occurs at NO concentration thresholds that have not been defined for EGFR activation in breast cancer. The article shows that aberrant iNOS expression and altered S-nitrosation in HER2-driven breast cancer can confer resistance to trastuzumab therapy. This study also mentions recent clinical trial designs combining NOS inhibitors (e.g., L-NMMA) and HER-targeted agents for the treatment of breast cancer. Moreover, the study exposes gaps in knowledge pertaining to lesser-studied HER family members and their nitrosative post-translational modifications, indicating promising areas for future research on the interplay between redox and oncogenesis.

NO biosynthesis extends beyond canonical NOS-dependent mechanisms, as is particularly evident in skeletal muscle physiology. Upanan et al. investigate compensatory nitrate–nitrite reductive pathways that provide alternative NO sources under conditions of nNOS deficiency or altered enzymatic activity. Dietary nitrate supplementation upregulates nitrate transporters (sialin and CLC1) and the nitrate reductase enzyme XOR, markedly increasing muscle nitrate reserves and maintaining NO signaling, which is critical for mitochondrial function and muscle performance. The authors show that the levels of protein and mRNA of eNOS or iNOS, and eNOS activation are not upregulated, indicating NO synthase-independent compensatory mechanisms.​ Interestingly, the muscle, but not the liver, of nNOS-deficient mice exhibits a pronounced induction of the nitrate–nitrite reductive pathway genes/proteins in response to nitrate overload, pinpointing tissue-specific adaptation in NO generation. Finally, nitrate supplementation does not increase the expression of inflammatory cytokine IL-6, suggesting that the nitrate–nitrite pathway dissociates from overt inflammatory signaling in muscle. These adaptive mechanisms, more robustly present in the muscle than in the liver, support muscle contractile function in genetic or disease states related to NOS impairment, laying the groundwork for the nutritional or pharmacological exploitation of enhancing endogenous NO availability to mitigate muscular dystrophies or metabolic disorders.

Cardiovascular studies further highlight NOS isoform-specific effects, notably in the context of erythropoietin (EPO)-mediated cardiac responses. Lee et al. deploy a multi-strain mouse model approach to dissect the contributions of tissue-specific and NOS isoforms to EPO-induced heart effects. They demonstrate that while acute EPO administration promotes cardiovascular protection, chronic high-dose EPO produces deleterious cardiac dysfunction dependent on nNOS activity and on EPO receptor expression in non-erythroid tissues. This dichotomy is revealed in transgenic mouse models, in which nNOS knockout confers cardioprotection against EPO-induced adverse effects despite preserved erythropoiesis. Interestingly, EPO treatment in nNOS-knockout mice improves glucose and insulin tolerance, revealing a separation between metabolic and cardiac EPO actions and providing new evidence for nNOS-specific metabolic regulation under EPO challenge.​ Akt phosphorylation (a key pro-survival and remodeling pathway) and Nox4 (a major source of cardiac reactive oxygen species) are upregulated by EPO in wild-type mice but not in nNOS-knockout mice, reinforcing the role of nNOS as a mediator of cardiac maladaptation under chronic EPO.​ Underlying modulation of heart failure-associated gene networks and signaling pathways, such as ERK and Akt, elucidates the nuanced interplay between NOS isoforms and cardiac remodeling. This supports a future where isoform-specific NO modulation optimizes therapeutic benefits while minimizing risks. Moreover, the identification of nNOS as essential to EPO-induced cardiac injury reveals a potential therapeutic target to decouple the hematopoietic benefits of EPO from its cardiovascular side effects and opens the door to exploring nNOS-linked protein S-nitrosylation in EPO biology.

Lewis et al. introduce technological advances that exploit endogenous NO pools, such as the use of infrared (IR) light to induce rapid, sustained vasodilation via NO release from endothelial stores and activation of soluble guanylyl cyclase pathways. This is significant work, since it has long been known that light can mediate vasodilation, but the wavelengths involved were thought to be in the UV range. The authors utilize a custom setup to deliver precise IR light pulses (at 1,460 nm, a wavelength not previously explored for direct vascular effects) to live, isolated rat occipital arteries mounted in wire myographs, allowing for direct spatial targeting and real-time tension recording.​ Their study combines pharmacological manipulation (e.g., endothelial denudation and the use of ODQ, a selective sGC inhibitor) with IR stimulation, rigorously isolating the contributions of the endothelium and NO/sGC/cGMP signaling to IR-induced vasodilation.​ This photobiomodulation effect, prominently seen in rat occipital arteries, implicates pre-formed S-nitrosothiol pools and endothelial vesicular release as mechanisms. Sustained post-irradiation vasodilation suggests that long-lived intracellular signaling cascades, including second messengers (e.g., cGMP and Ca^2+^) and protein nitrosation/phosphorylation, contribute to vascular tone regulation beyond immediate stimuli. The reported vasodilatory effects of IR persist well beyond the exposure period, suggesting that short flashes of light can sustain improved vascular function for a longer duration than traditional pharmacological interventions.​ These findings hold clinical promise for the non-invasive treatment of vascular and neural disorders, with IR light offering precise temporal and spatial control of NO-mediated vasoreactivity. This makes these energy-based therapies promising for restoring targeted blood flow in disease contexts such as hypertension and diabetes.​ Finally, the segment-specific vascular responses revealed may inform future precision vascular interventions or neuromodulation strategies using IR light, especially in tissues or organs with unique endothelial architecture.​

This integrative collection confirms NO and RNS as nodal regulators that span metabolism, immunity, oncogenesis, and tissue physiology, with interdependencies shaped by concentration, compartmentalization, and temporal dynamics. Translationally, these insights predict promising avenues toward redox-targeted therapies, dietary modulation, and biophysical interventions. Looking forward, several trajectories may be taken: the refinement of spatial-temporal NO imaging to resolve subcellular signaling gradients; the dissection of redox-driven post-translational modifications in disease contexts; the exploitation of isoform-specific NOS modulation; and the harnessing of endogenous NO metabolite pools for innovative therapies. Advancements in measurement technologies, bioinformatic integration, and multi-omics promise to unravel NO-related signaling networks with greater clarity, fueling precision medicine approaches that are tailored to the mechanisms of redox biology. Ultimately, this body of work elevates NO from a mere diffusible mediator to a master integrator of physiological homeostasis and disease modulation, offering a framework for bridging molecular discovery and clinical transformative potential. Collectively, these findings usher in an era where NO-centric strategies will reshape the landscape of inflammation, cancer, cardiovascular, and metabolic therapeutics, fulfilling the vision of translating elegant redox chemistry into robust clinical impact.
